# The Human Host Defense Ribonucleases 1, 3 and 7 Are Elevated in Patients with Sepsis after Major Surgery—A Pilot Study

**DOI:** 10.3390/ijms17030294

**Published:** 2016-02-26

**Authors:** Lukas Martin, Patrick Koczera, Nadine Simons, Elisabeth Zechendorf, Janine Hoeger, Gernot Marx, Tobias Schuerholz

**Affiliations:** Department of Intensive Care and Intermediate Care, University Hospital Aachen, Rheinisch-Westfälische Technische Hochschule (RWTH) Aachen University, Pauwelsstrasse 30, Aachen 52074, Germany; lmartin@ukaachen.de (L.M.); pkoczera@ukaachen.de (P.K.); nsimons@ukaachen.de (N.S.); ezechendorf@ukaachen.de (E.Z.); jhoeger@ukaachen.de (J.H.); gmarx@ukaachen.de (G.M.)

**Keywords:** human RNases, host-defense protein, sepsis

## Abstract

Sepsis is the most common cause of death in intensive care units and associated with widespread activation of host innate immunity responses. Ribonucleases (RNases) are important components of the innate immune system, however the role of RNases in sepsis has not been investigated. We evaluated serum levels of RNase 1, 3 and 7 in 20 surgical sepsis patients (Sepsis), nine surgical patients (Surgery) and 10 healthy controls (Healthy). RNase 1 and 3 were elevated in Sepsis compared to Surgery (2.2- and 3.1-fold, respectively; both *p* < 0.0001) or compared to Healthy (3.0- and 15.5-fold, respectively; both *p* < 0.0001). RNase 1 showed a high predictive value for the development of more than two organ failures (AUC 0.82, *p* = 0.01). Patients with renal dysfunction revealed higher RNase 1 levels than without renal dysfunction (*p* = 0.03). RNase 1 and 3 were higher in respiratory failure than without respiratory failure (*p* < 0.0001 and *p* = 0.02, respectively). RNase 7 was not detected in Healthy patients and only in two patients of Surgery, however RNase 7 was detected in 10 of 20 Sepsis patients. RNase 7 was higher in renal or metabolic failure than without failure (*p* = 0.04 and *p* = 0.02, respectively). In conclusion, RNase 1, 3 and 7 are secreted into serum under conditions with tissue injury, such as major surgery or sepsis. Thus, RNases might serve as laboratory parameters to diagnose and monitor organ failure in sepsis.

## 1. Introduction

Sepsis is a common syndrome in intensive care units (ICUs) worldwide and is associated with high morbidity, mortality and long-term disability [1]. Similar to severe trauma, burn or other overwhelming stresses, the underlying pathophysiology is characterized by the widespread activation of host innate immune responses [2]. These complex mechanisms lead to an excessive inflammatory response resulting in the impaired function of several organs [3].

In the beginning of the 20th century antimicrobial peptides (AMPs) were found to be important players of the innate immune system. Thereby, AMPs interact with a broad spectrum of invading pathogens of different species such as bacteria, viruses, fungi as well as parasites [4,5]. Today, over 2600 natural occurring AMPs have been described not only targeting invading pathogens, but also playing a central role in recruiting and promoting elements of the innate immune system [6]. The mammalian ribonucleases (RNases) are found in several secretions playing a role in host defense, such as milk, seminal fluid, saliva and tears [7]. Beside these mucosal secretions, a wide range of mammalian tissues express RNases, for example brain [8], liver [9] and kidney [10]. In addition, RNases are secreted by a variety of immune cells, including eosinophils [11,12], neutrophils [13], monocytes [14] as well as macrophages [15]. The most well-characterized member of the RNase superfamily is RNase A, which has been the subject of intense biochemical study for over half a century [7]. This superfamily consists of eight canonical members: RNase 1 (pancreatic RNase), RNase 2 (eosinophil-derived cationic protein, EDN), RNase 3 (eosinophil cationic protein, ECP), RNase 4, RNase 5 (angiogenin), RNase 6, RNase 7 (skin derived RNase) and RNase 8 [7,16,17].

Despite the name *pancreatic* RNase, RNase 1 is expressed in a variety of tissues, including human endothelial cells [18]. Thus, besides its importance as a digestive enzyme, the wide extracellular distribution of RNase 1 suggests the involvement in systemic processes, in particular the regulation of vascular homeostasis [17,18]. Due to its remarkable catalytic activity, RNase 1 acts as a potent nonspecific scavenger of pathogenic RNA molecules [19]. Furthermore, RNase 1 is likely to activate human dendritic cells, leading to the production of a variety of soluble pro-inflammatory mediators including cytokines, chemokines, growth factors as well as soluble receptors [20].

RNase 3, which is also known as ECP, is found in the secondary granules of eosinophils [21]. For several years now, RNase 3 has been associated with a variety of *chronic* inflammatory disorders, such as bronchial asthma [22] and Crohn´s disease [23]. In this context, RNase 3 levels are currently used as a clinical marker for the diagnosis and progression of *chronic* diseases, however the physiological significance of this has not yet been elucidated [7]. It has been demonstrated, that RNase 3 possesses anti-bacterial, anti-helminthic as well as anti-viral activity [7]. Similar to other AMPs, the anti-bacterial RNase 3 exhibits its anti-bacterial properties in both, bactericidal disruption of the outer and inner membrane of the bacteria [24] as well as the binding of their constitutively released cell wall compounds (*i.e*., lipopolysaccharide or peptidoglycan) [25]. In addition, RNase 3 inhibits mammalian cell growth by exhibiting cytotoxic [26] and pro-apoptotic activity [27], however the exact nature and molecular basis of its cytotoxic activity are not fully understood [7].

RNase 7 was first identified as the most present RNase of human skin and secreted by keratinocytes [28,29]. However, its presence is likely to be beyond the skin since RNase 7 is expressed in epithelial tissues involved in host defense, such as the respiratory or urinary tract including the kidney [30,31]. RNase 7 possesses anti-bacterial activity against a wide range of bacteria, including vancomycin-resistant *Enterococcus faecium* [28]. Thereby, similar to RNase 3 and other AMPs, the anti-bacterial activity consists of both, bactericidal membrane disruption as well as binding and neutralizing of highly immune-potent circulating cell wall compounds [32,33].

However, despite the knowledge that sepsis can be considered a race to the death between pathogens and the host immune system [34] as well as the intense study of structure and activity of RNases, data on the roles of RNases in sepsis are currently completely lacking. Thus, the aim of our study was to investigate serum levels of RNase 1, 3 and 7 to verify the secretion of RNAses in response to acute systemic infection such as sepsis in surgical patients.

## 2. Results and Discussion

### 2.1. Study Population

The characteristics of the study population according to the groups (Healthy, Surgery, Sepsis) are shown in Table 1. The patients (59% male) had a mean age of 63 (52–75) (median, interquartile range (IQR)) years, with no significant difference in age between the groups. Patients with sepsis had significant higher lengths of stay (LOS) in the ICU (7 (5–11) *vs*. 1 (1–3) days; median (interquartile range, IQR), *p* < 0.0001) as well as a significant higher Acute Physiology and Chronic Health Evaluation II score (APACHE II) (11.5 (8.5–16.8) *vs*. 9 (4.0–10.5); *p* = 0.03), compared to surgical ICU patients without sepsis (Table 1).

### 2.2. Serum Levels of RNases

**Figure 1:**
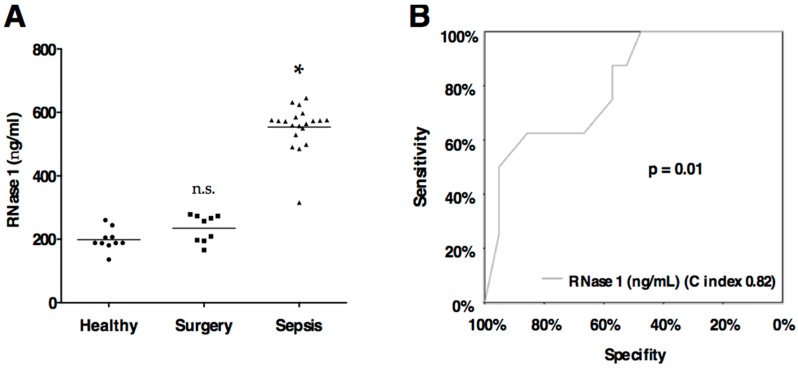
(**A**) RNase 1 serum levels. Serum levels of ribonuclease 1 (RNase 1) are displayed according to the groups. Black line indicates the mean. Groups: Healthy subjects (*n* = 10), surgical ICU patients (Surgery, *n* = 9) and surgical ICU patients with sepsis (*n* = 20) according to the definition published by the Surviving Sepsis Campaign [40]. * *p* < 0.05 *vs.* Healthy; n.s., non significant *vs.* Healthy; (**B**) RNase 1 predicts development of more than two organ dysfunctions according to the definition published by the Surviving Sepsis Campaign [40]. Logistic regression was used to evaluate serum levels of RNase 1 for the prediction of the development of more than two organ dysfunctions, and receiver operating characteristic (ROC) curves were constructed for illustration.

**Figure 2:**
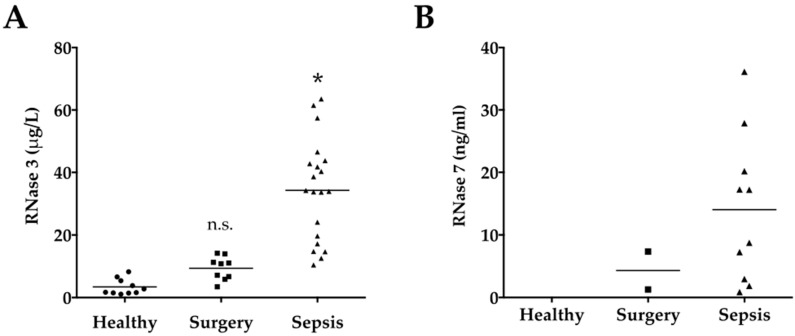
(**A**) RNase 3 serum levels. Serum levels of ribonuclease 3 (RNase 3) are displayed according to the groups; (**B**) RNase 7 serum levels. Serum levels of ribonuclease 7 (RNase 3) are displayed according to the groups. RNase 7 was not detected in Healthy, only in two patients of Surgery and in 10 of 20 sepsis patients. Black line indicates the mean. Groups: Healthy subjects (*n* = 10), surgical ICU patients (Surgery, *n* = 9) and surgical ICU patients with sepsis (*n* = 20) according to the definition published by the Surviving Sepsis Campaign [40]. * *p* < 0.05 *vs.* Healthy; n.s., non significant *vs.* Healthy.

Linear regression including age, gender and BMI revealed no significant association with RNase 1, 3, and 7 levels (all *p* > 0.05). Serum levels of RNase 1 and 3 were significantly elevated in Sepsis compared to Surgery or Healthy (all *p* < 0.0001; Table 2, Figures 1A and 2A). RNase 7 was not detected in Healthy and only in two patients of Surgery, however RNase 7 was determined in 10 of 20 sepsis patients (Table 2, Figure 2B).

### 2.3. RNase 1

Endothelial cells express and release RNase 1 [7], which consequently circulates in blood. The reported concentrations (300–400 ng/mL) [18,35] are comparable to our measured levels in healthy human subjects (Table 2; Figure 1A). Sepsis and major surgery are conditions of tissue injury, which result in the release of extracellular RNA (eRNA). As a danger molecule eRNA induces coagulation [36], endothelial hyperpermeability [37] as well as the release of pro-inflammatory cytokines (*i.e*., tumor necrosis factor alpha (TNF-α) or interleukin 1β (IL-1 β)) [38]. Thereby, RNase 1 serves as a natural blood vessel-protective antagonist of eRNA [39]. All of these pathological conditions contribute to organ dysfunction and multi organ failure in sepsis [3]. In our study, eight of 29 included patients (27.6%) developed more than two organ dysfunctions according to the definition published by the Surviving Sepsis Campaign [40]. Furthermore, RNase 1 showed a high predictive value for the development of more than two organ failures (AUC 0.82, *p* = 0.01; Figure 1B).

**Figure 3:**
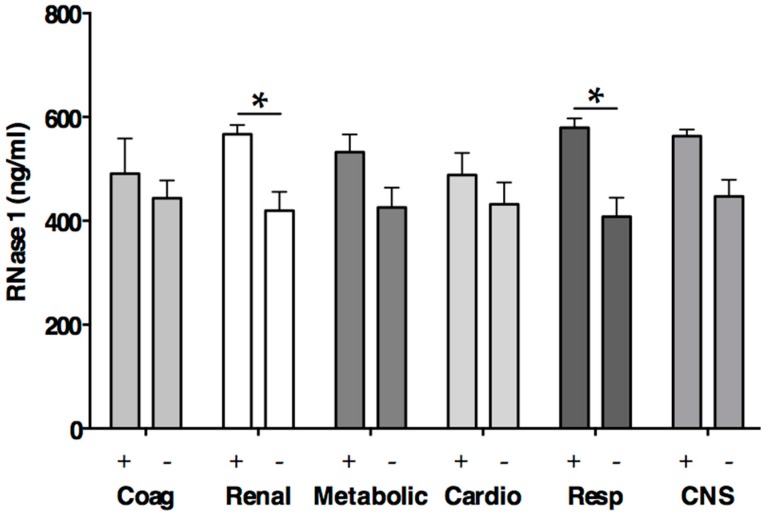
RNase 1 levels according to organ dysfunctions defined by the Surviving Sepsis Campaign [40]. Mean ± standard deviation. * *p* < 0.05; Coag Coagulation; Cardio Cardiovascular; Resp Respiratory; CNS Central nervous system; Plus (+) denotes presence of organ dysfunction; Minus (−) denotes absence of organ dysfunction.

Already in 1977, a study on patients with leukemia established an association of RNase 1 serum levels with the development renal insufficiency and a positive correlation with serum creatinine [41]. Similarly, in our study, patients with renal dysfunction revealed significantly higher RNase 1 levels compared to patients without renal dysfunction (575.8 (529.3–597.6) *vs*. 492.1 (264.1–573.3) ng/mL, *p* = 0.03, Figure 3); however linear regression of serum creatinine did not reveal a significant association with RNase 1. Besides renal dysfunction, patients with respiratory failure had significantly higher RNase 1 levels compared to patients without respiratory failure (580.6 (540.9–623.4) *vs*. 485.4 (261.9–568.1) ng/mL, *p* < 0.0001, Figure 3). Thus, in the early phase of sepsis, RNase 1 seems to be upregulated in conditions with tissue injury. Probably, RNase 1 thereby acts as a host protective antagonist of eRNA, however further studies are needed to clarify the molecular role of RNase 1 in sepsis. The RNase response may differ in the later stages of sepsis, since *in vitro* data indicate that pro-inflammatory cytokines, significantly decrease the cellular expression and release of RNase 1 when administered to human umbilical vein endothelial cells for 48 h [39].

### 2.4. RNase 3

Eosinophils, the major source of RNase 3, plays a protective role in the innate immune response to sepsis [42]. Recent data indicate that sepsis and bacterial infections are associated with a marked eosinopenia [43]. In contrast, we measured no significant difference in the eosinophil count between Surgery and Sepsis (Table 1). This can be explained by the more common development of eosinopenia in sepsis during the later stages of sepsis [43]. Nevertheless, the total eosinophil count is not solely decisive for the amount of RNAse 3. The generation of RNAse 3 may be opposed by the production of eosinophil protein x/eosinophil-derived neurotoxin (EPX/EDN, RNase 2) [44].

**Figure 4:**
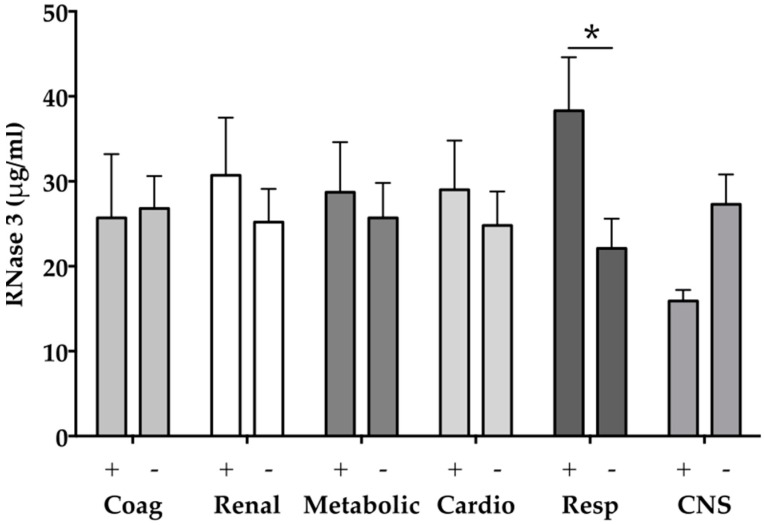
RNase 3 levels according to organ dysfunctions defined by the Surviving Sepsis Campaign [40]: * *p* < 0.05; Coag Coagulation; Cardio Cardiovascular; Resp Respiratory; CNS Central nervous system; Plus (+) denotes presence of organ dysfunction; Minus (−) denotes absence of organ dysfunction.

However, our data indicate significantly elevated levels of RNase 3 in patients with sepsis compared to healthy volunteers or surgical ICU patients (all *p* < 0.001; Table 1, Figure 2). Eosinophil granulocytes especially are relatively abundant in the respiratory tract mucosa [45]. Serum levels of RNase 3 are significantly elevated in children with Mycoplasma pneumonia and directly correlate with the presence of eosinophilia (*r* = 0.349) [46]. In the current study, eight of 29 included patients (27.6%) developed respiratory failure according to the definition published by the Surviving Sepsis Campaign [40]. Notably, patients with respiratory failure showed significantly higher circulating RNase 3 levels compared to patients without respiratory failure (36.4 (21.3–56.8) *vs*. 14.2 (10.6–37.1) ng/mL, *p* = 0.02, Figure 4). Thus, our data might indicate for the first time a role of RNase 3 in sepsis associated respiratory failure. However, further studies are needed to clarify the pro- and anti-inflammatory activity of RNase 3 in sepsis associated lung infection. Until then, it remains unknown whether RNase 3 plays a role by facilitating the immune response at the onset of infection or by killing the pathogen and the removal of damaged cells in later stages of infection [7].

### 2.5. RNase 7

**Figure 5:**
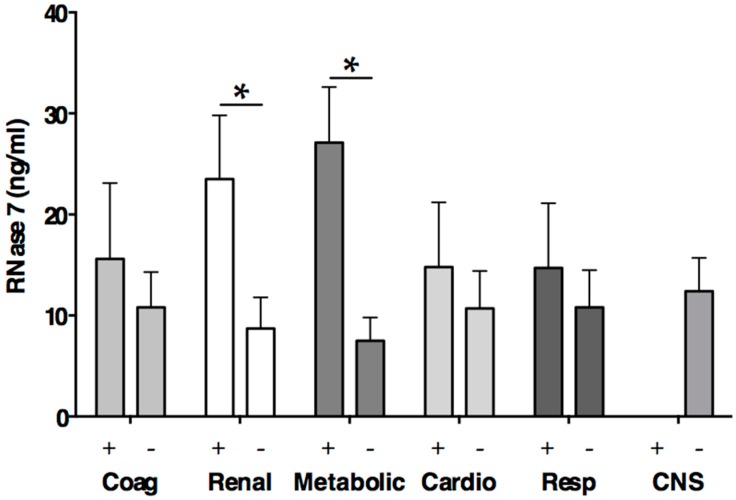
RNase 7 levels according to organ dysfunctions defined by the Surviving Sepsis Campaign [40]. * *p* < 0.05; Coag Coagulation; Cardio Cardiovascular; Resp Respiratory; CNS Central nervous system; Plus (+) denotes presence of organ dysfunction; Minus (−) denotes absence of organ dysfunction.

RNase 7 is produced by the urothelium as well as by intercalated cells of the kidney. Thereby, it helps to maintain the number of microbes in the human urinary tract at low levels [10]. Recent data indicated that even transient impairment of renal function in patients with sepsis increases the risk of death [47]. Thus, the early detection of renal dysfunction is crucial for the outcome of the patients. We detected in seven of 29 included patients (24.1%) a renal dysfunction defined by the Surviving Sepsis Campaign [40]. RNase 7 levels revealed significantly higher levels in patients with renal dysfunction compared to patients without renal dysfunction (17.3 (17.2–36.1) *vs*. 7.3 (1.6–14.5) ng/mL, *p* = 0.04, Figure 5). However, linear regression of serum creatinine did not reveal a significant association with RNase 7. Recently, a study on human urinary tract infection reported increased RNase 7 concentrations in infected urine as well as an increase in RNase 7 expression in the kidney and urinary tract in response to infection [31]. Beside the urine analysis, levels of RNase 7 were also investigated in blood, serum, and plasma. Notably, except for the detection in urine RNase 7 was not detected in the investigated samples [31]. Using a highly sensitive commercial ELISA with a minimum detectable value of 0.58 ng/mL RNase 7, we detected RNase 7 in 10 of 20 sepsis patients, however RNase 7 was not detected in Healthy and only in two patients of Surgery (Table 2, Figure 2B). Thus, the relevant secretion of RNase 7 in serum seems to be restricted to conditions with pronounced tissue injury, such as major surgery or sepsis. Furthermore, we detected a significant difference of RNase 7 serum levels according to the presence or absence of metabolic failure defined by the Surviving Sepsis Campaign [40] (27.9 (17.3–36.1) *vs*. 7.3 (1.6–13.0) ng/mL, *p* = 0.02, Figure 5). These findings support the hypothesis that RNase 7 is secreted into the serum during systemic injury, since metabolic failure represents a common reflection of tissue hypoperfusion and injury [48].

## 3. Materials and Methods

### 3.1. Ethical Approach

The patients or next of kin gave written informed consent before any study related procedure. The study and related procedure were approved by the ethics committee of the University Hospital Aachen under EC Nr. 206_09, 5 January 2010. This study was conducted in accordance with the Declaration of Helsinki in its actual form. The samples were obtained from the RWTH centralized Biomaterial Database (RWTH cBMB; University Hospital RWTH, Aachen, Germany).

### 3.2. Study Population

We prospectively included 29 surgical ICU patients at the Department of Intensive Care and Intermediate Care of the University Hospital Aachen (Aachen, Germany) between September 2012 and March 2014. Twenty of those patients suffered from sepsis according to the sepsis definitions of the Surviving Sepsis Campaign (SCCM) [40]. The exclusion criteria were age <18 years old, recent organ transplantation, pregnancy, heart surgery or receiving palliative care. Furthermore, we included 10 healthy human individuals as control. Organ dysfunction (Coagulation, renal, metabolic, cardiovascular, respiratory or central venous system) were defined according to the definition published by the Surviving Sepsis Campaign [40].

### 3.3. Data Collection

All relevant data were extracted from medical records and electronic bedside flow charts (IntelliSpace Critical Care and Anesthesia (ICCA); Philips Healthcare, Andover, MA, USA).

### 3.4. Sample Collection

In accordance to the study and ethical protocol, all blood samples of surgical ICU patients were drawn from an already inserted intravascular catheter. Blood samples were taken during the first 18 h after the onset of sepsis defined by the Surviving Sepsis Campaign [40]. Healthy controls consented to a single peripheral blood sample. Serum samples were allowed to clot at room temperature for 30 min and were centrifuged at 2000× *g* for 10 min.

### 3.5. Ribonucelases–Enzyme-Linked Immunosorbent Assays

The amounts of RNases in serum were determined using commercially available ELISAs (all Cloud-Clone Corp., Houston, TX, USA) according to the manufacturer’s instructions. Briefly, a total of 100 µL of standards or samples were added to the wells followed by the addition of 100 µL detection reagent A. Serum was diluted for the measurements of RNase levels (RNase 1 [1:1000]; RNase 3 [1:200]; RNase 7 [1:2]). The plate was incubated for 1 h at 37 °C. After three wash steps with the supplied wash solution, 100 µL detection reagent B was added to each well. The plate was incubated for 30 min at 37 °C. 90 µL substrate solution was added to each well before the reaction was halted with stop solution after 10 min. The absorbance was measured at 450 nm on a microplate reader (Sunrise Tecan, Crailsheim, Germany). The lower detection limit of the immunoassay for RNase 1, 3 and 7 was 30 pg/mL, 16 pg/mL and 0.58 ng/mL, respectively.

### 3.6. Statistical Analyses

Normal or non- normal data distribution has been verified before evaluating differences between the groups. Kruskal-Wallis test was used for continuous variables and Chi-square test for categorical variables. Accordingly, values are expressed as mean and standard deviation (SD), median and interquartile range (IQR) or as count and percentage (%), as appropriate. Logistic regression was used to evaluate serum levels of RNases for the prediction of organ dysfunction, and receiver operating characteristic (ROC) curves were constructed for illustration. The area under the ROC curve (AUC, or C index) was given as an effect measurement. All of the statistical tests were 2-tailed, and a two-sided *p*-value of 0.05 was considered to be significant. The statistical analyses were performed using IBM SPSS Statistics 22.1 (IBM, New York, NY, USA) and GraphPad Prism Version 5.01 (Graphpad Software, San Diego, CA, USA).

## Figures and Tables

**Table 1:** Patients’ characteristics.

	Healthy (*n* = 10)	Surgery (*n* = 9)	Sepsis (*n* = 20)	*p*-Value
Age (years) (IQR)	55 (49–74)	70 (55–75)	64 (55–75)	0.63
Male sex (%)	7 (70.0)	4 (44.4)	12 (60.0)	0.52
BMI (kg/m^2^) (IQR)	-	24.3 (22.1–35.1)	27.3 (30.9–22.6)	0.72
Diabetes mellitus (%)	-	3 (33.3)	4 (20.0)	0.5
Creatinine (mg/dL) (IQR)	-	0.9 (0.6–1.0)	0.8 (0.7–1.3)	0.71
Hemoglobin (g/dL) (IQR)	-	11.3 (10.1–12.0)	9.3 (8.4–9.6)	<0.0001
Platelets (10^9^ cells/nL) (IQR)	-	255.0 (168.0–296.0)	193.5 (173.0–257.8)	0.32
White cells (10^9^ cells/nL) (IQR)	-	10.5 (8.8–12.5)	17.5 (14.3–21.1)	0.02
Neutrophil (10^9^ cells/nL) (IQR)	-	85.5 (84.9–87.9)	86.0 (76.5–93.0)	0.89
Eosinophil (10^9^ cells/nL) (IQR)	-	0.2 (0.1–0.2)	0.0 (0.0–1.5)	0.69
Monocyte (10^9^ cells/nL) (IQR)	-	4.9 (2.5–6.4)	4.3 (3.5–6.0)	0.91
Lymphocyte (10^9^ cells/nL) (IQR)	-	9.3 (4.1–11.7)	5.0 (2.9–10.5)	0.26
Albumin (g/L) (IQR)	-	31.0 (26.5–38.5)	22.0 (19.0–26.0)	<0.0001
PCT (ng/mL) (IQR)	-	0.1 (0.0– 0.2)	3.1 (0.6–21.8)	<0.0001
CRP (mg/dL) (IQR)	-	6.6 (2.8– 25.0)	170.5 (113.0–230.0)	<0.0001
Lactate (mmol/L) (IQR)	-	0.9 (0.8–1.7)	1.5 (1.2–2.5)	0.05
Fluid administration within first 24 h (L) (IQR)	-	4.9 (3.0–5.0)	5.2 (3.5–7.0)	0.13
Urine output within first 24 h (L) (IQR)	-	1.9 (1.3–2.3)	3.4 (2.6–4.3)	<0.0001
SOFA (points) (IQR)	-	5.0 (0.5–6.0)	6.0 (3.3–8.0)	0.06
APACHE II (points) (IQR)	-	9.0 (4.0–10.5)	11.5 (8.5–16.8)	0.03
Vasopressors (h) (IQR)	-	2.5 (0.0–31.0)	30.0 (2.0–68.8)	0.11
MV (h) (IQR)	-	3.0 (0.3–7.0)	3.0 (0.0–45.5)	0.55
LOS ICU (days) (IQR)	-	1.0 (1.0–3.0)	7.0 (5.0–12.0)	<0.0001
28-day mortality (%)	-	0 (0.0)	0 (0.0)	-

Categorical and continuous variables are presented as n (%) and median (interquartile ranges, IQR), respectively. Kruskal-Wallis test was used to compare categorical and continuous variables, respectively. BMI, body-mass-index; PCT, Procalcitonin; CRP, C-reactive protein, SOFA, Sequential Organ Failure Assessment score; APACHE II, Acute Physiology and Chronic Health Evaluation II score; MV, mechanical ventilation; LOS, length of stay.

**Table 2:** Serum Levels of RNases.

	Healthy (*n* = 10)	Surgery (*n* = 9)	Sepsis (*n* = 20)	*p*-Value
RNase 1 (ng/mL) (IQR)	188.7 (188.0–216.2)	257.5 (196.2–273.3)	572.6 (534.5–582.9)	<0.0001
RNase 3 (μg/mL) (IQR)	2.2 (1.5–5.7)	10.8 (6.3–12.6)	34.1 (17.8–43.5)	<0.0001
RNase 7 (ng/mL) (IQR)	n.d.	4.3 (1.0–7.3) ^#^	13.0 (2.7–22.1) ^##^	0.28

Categorical and continuous variables are presented as n (%) and median (interquartile range, IQR), respectively. Kruskal-Wallis test was used to compare categorical and continuous variables, respectively. n.d., not detected; ^#^ only detected in 2/9 patients; ^##^ only detected in 10/20 patients.

## Abbreviations

AMP	antimicrobial peptide
APACHE II	Acute Physiology and Chronic Health Evaluation II score
BMI	body-mass-index
CRP	C-reactive protein
ECP	eosinophil cationic peptide
EDN	eosinophil-derived cationic protein
ELISA	enzyme linked immunoassay
ICU	intensive care unit
IL-1 β	interleukin 1β
LOS	length of stay
MV	mechanical ventilation
PCT	procalcitonin
RNase	ribonucleases
SCCM	Society of Critical Care Medicine
SOFA	Sequential Organ Failure Assessment score
TNF-α	tumor necrosis factor alpha
